# Dynamic development of the first synapse impinging on adult-born neurons in the olfactory bulb circuit

**DOI:** 10.1186/2042-1001-1-6

**Published:** 2011-02-01

**Authors:** Hiroyuki Katagiri, Marta Pallotto, Antoine Nissant, Kerren Murray, Marco Sassoè-Pognetto, Pierre-Marie Lledo

**Affiliations:** 1 Laboratory for Perception and Memory, Institut Pasteur, Paris, France; 2 Centre National de la Recherche Scientifique (URA 2182), 25 rue du Dr. Roux, F-75724 Paris, France; 3 Department of Anatomy, Pharmacology and Forensic Medicine and National Institute of Neuroscience-Italy, University of Turin, Italy

## Abstract

The olfactory bulb (OB) receives and integrates newborn interneurons throughout life. This process is important for the proper functioning of the OB circuit and consequently, for the sense of smell. Although we know how these new interneurons are produced, the way in which they integrate into the pre-existing ongoing circuits remains poorly documented. Bearing in mind that glutamatergic inputs onto local OB interneurons are crucial for adjusting the level of bulbar inhibition, it is important to characterize when and how these inputs from excitatory synapses develop on newborn OB interneurons. We studied early synaptic events that lead to the formation and maturation of the first glutamatergic synapses on adult-born granule cells (GCs), the most abundant subtype of OB interneuron. Patch-clamp recordings and electron microscopy (EM) analysis were performed on adult-born interneurons shortly after their arrival in the adult OB circuits. We found that both the ratio of *N*-methyl-D-aspartate receptor (NMDAR) to α-amino-3-hydroxy-5-methyl-4-isoxazolepropionic acid receptor (AMPAR), and the number of functional release sites at proximal inputs reached a maximum during the critical period for the sensory-dependent survival of newborn cells, well before the completion of dendritic arborization. EM analysis showed an accompanying change in postsynaptic density shape during the same period of time. Interestingly, the latter morphological changes disappeared in more mature newly-formed neurons, when the NMDAR to AMPAR ratio had decreased and functional presynaptic terminals expressed only single release sites. Together, these findings show that the first glutamatergic inputs to adult-generated OB interneurons undergo a unique sequence of maturation stages.

## Background

Adult neurogenesis, a process encompassing the generation, maturation and synaptic integration of new neurons in the adult brain, represents a striking form of structural adult neural plasticity [1-3]. In the adult olfactory bulb (OB), nearly all newly recruited neurons become local interneurons [4]. About 95% of the new cells differentiate into granule cells (GCs) and less than 4% become periglomerular cells [5,6], with glutamatergic juxtaglomerular neurons accounting for the rest [7]. The newly-formed interneurons project dendrites that establish synaptic contacts with pre-existing partners, becoming indistinguishable from other mature interneurons within few weeks [8-13].

**Figure 1:**
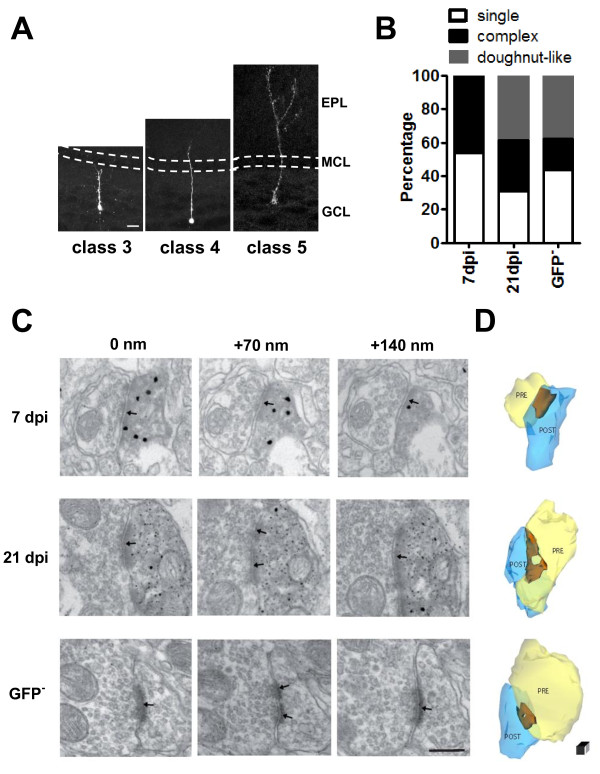
**Changes in synapse morphology during newborn granule cell **(**GC) maturation**. **(A) **Adult-born GCs of the olfactory bulb (OB) labeled by injection of biocytin. Cell maturation is characterized by progressive growth of the apical dendrite toward the external plexiform layer (EPL). GCL = granule cell layer; MCL = mitral cell layer. Scale bar = 20 μm. **(B) **Synapses with a single homogeneous postsynaptic density (PSD) were prevalent in GCs at 7 days post-injection (dpi), whereas older cells exhibited a larger percentage of synapses with a more complex morphology including a doughnut-like shape. **(C) **Representative images of asymmetric synapses between axonal profiles and dendrites of 7 dpi, 21 dpi and pre-existing (green fluorescent protein (GFP)-negative) GCs (three sections of each series are shown with depth). The dendrites of 7 dpi and 21 dpi GCs are identified by GFP immunolabeling. Arrows identify PSD. Scale bar = 200 nm. **(D) **Three-dimensional reconstruction of synapses based on axonal profiles (PRE = light yellow) with GC dendrites (POST = transparent blue), revealing the variable shape of the synaptic junctions (brown). The synapse of a 7 dpi GC shows a single, compact PSD. Synapses of 21 dpi and GFP^- ^GC have a doughnut-like morphology. Scale **bar = **50 nm.

**Figure 2:**
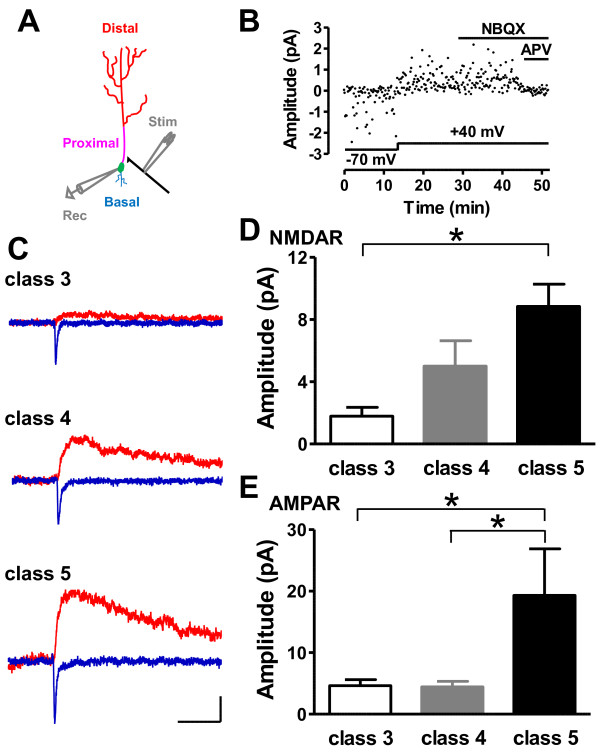
**Developmental change in glutamatergic response at the proximal site**. **(A) **Schematic diagram of experimental procedures. Three domains of dendritic regions of axonless GCs are divided (distal dendrite = red, proximal dendrites = pink, basal dendrites = blue). Whole-cell recordings (gray, *Rec*) were performed from soma of GFP-positive GCs (green) and the stimulating electrode (gray, *Stim*) activated centrifugal fibers (black). **(B) **Typical plot of response amplitude by minimal stimulation. Membrane potential is shown below and drug application above the plot. **(C) **Representative average traces. 3-hydroxy-5-methyl-4-isoxazolepropionic acid receptor (AMPAR)-mediated excitatory postsynaptic currents (EPSCs) (blue) were recorded at the holding potential of -70 mV in the presence of 10 μmol/l SR-95531 (gabazine). *N*-methyl D-aspartate (NMDAR)-mediated EPSCs (red) were recorded at +40 mV in the presence of a combination of 10 μmol/l SR-95531 and 10 μmol/l 2,3-dioxo-6-nitro-1,2,3,4-tetrahydrobenzo[f]quinoxaline-7-sulfonamide (NBQX). Scale bar = 50 ms and 2.5 pA. **(D) **NMDAR-mediated EPSCs gradually increased during maturation (class 3 versus class 5; *P < 0.05) (class 3: six slices, six mice; class 4: nine slices, eight mice; class 5: four slices, four mice). (E) The amplitude of AMPAR-mediated EPSCs was much higher in class 5 than in class 4 cells (class 3 versus class 4, class 3 versus class 5; *P < 0.05) (class 3: twelve slices, eleven mice; class 4: sixteen slices, fourteen mice; class 5: eleven slices, eight mice).

GCs are the largest population of interneurons in the bulb, outnumbering principal neurons by 100 to one [14]. Strikingly, they are also the most abundant subtype of neurons added to the adult OB [15]. GCs bear several short basal dendrites and one apical dendrite, which consists of a proximal domain and a branched distal domain (Figure 1A, Figure 2A). GCs form γ-aminobutyric acid (GABA)ergic output synapses onto the principal neurons, the mitral cells/tufted cells (MC/TCs), through dendrodendritic reciprocal contacts in the external plexiform layer [14]. In turn, they receive spatially segregated glutamatergic inputs at distinct dendritic sites [13,16,17]. Reciprocal dendrodendritic synapses with the MCs are the main source of distal glutamatergic input [18-20]. Whereas TC axon collaterals are superficially distributed in the internal plexiform layer, MC axon collaterals provide proximal glutamatergic inputs [20,21]. In addition, the somata and proximal domain of GC dendrites are the major targets of centrifugal fibers derived from several brain areas [22-24]. Interestingly, tetanic stimulation of centrifugal fibers can result in a long-lasting increase in excitatory synaptic transmission at the proximal synapses of GCs [25,26]. This ability to undergo synaptic plasticity is present in young rodent pups [25], and is only seen in adult-generated, developing interneurons in older animals [26]. As developing newborn GCs integrate into pre-existing OB circuits, they receive early excitatory proximal inputs that can profoundly influence their final stages of development, including the feed-forward inhibition they provide to their postsynaptic targets. However, how these early synaptic inputs on new neurons form and mature over time is still unknown.

In this study, we focused on the excitatory proximal inputs to newborn GCs. Pioneering studies on embryonal and postnatal development have shown that the first contacts to be formed are MC-GC dendrodendritic synapses at embryonic day (E)17. The formation of these first synaptic contacts is followed by the appearance of MC-GC axodendritic synapses at E18 [27,28]. Thus, key synapses important for the OB functioning are already present at birth, when olfaction is crucial for the survival of rodent pups. In contrast to embryonic synaptogenesis, we found that axodendritic proximal synapses were the first synaptic contacts formed on developing new GCs, shortly after the newcomer migrates radially into the bulb. Using electrophysiological and morphological approaches, we demonstrated that proximal synaptic inputs on newborn GCs are functionally developed and profoundly evolved over time much before the appearance of their output synapses. The overall developmental pattern of the proximal inputs on newborn GCs may be essential to shape the functional impact of the adult neurogenesis and, as a consequence, for proper circuit function.

## Results

### Structural reorganization of the glutamatergic synapses during GC maturation

Previous morphological studies have shown that adult-born GCs can be categorized into five distinct classes [29]. To characterize the early steps of synaptic integration, we focused on class 3 to class 5 neurons. We thus excluded migrating neurons (class 1 and 2) from the present analysis, as these cells do not exhibit direct synaptic activity (data not shown). We injected a green fluorescent protein (GFP)-encoding lentiviral vector into the rostral migratory stream (RMS) to label migrating neuroblasts [12,30]. At 3 days post-injection (dpi) of viruses, most GFP-positive cells were still navigating in the RMS, but a small proportion had already reached the bulb class 3 neurons (Figure 1A; see [30]). Their apical dendrites first reached the external plexiform layer a few days later (class 4). This step was followed by the formation of lateral branches with spines (class 5) (Figure 1A).

To examine early glutamatergic inputs contacting newborn GCs, we used pre-embedding immunolabeling for GFP combined with electron microscopy (EM). This ultrastructural analysis was performed in the deep portion of the GC layer, thus excluding TC axons located in the internal plexiform layer. At 7 dpi, EM analysis showed GFP-positive cells in the GC layer receiving asymmetric contacts from axon terminals. Serial sectioning revealed that some synapses at 7 dpi had a simple shape with a compact and homogeneous post-synaptic density (PSD) (7 over 13 sections) (Figure 1B, single cell; Figure 1C), whereas other synaptic junctions exhibited discontinuities of the PSD (6 over 13 sections; not shown). Synapses impinging onto more advanced maturing cells (21 dpi) and pre-existing GFP-negative cells exhibited more complex postsynaptic profiles, most notably, a doughnut-like shape with the PSD forming an almost complete ring around the central 'hole' (Figure 1B) (21 dpi cells: 5 over 13 sections; GFP-negative cells: 6 over 16 sections). Despite changes in the shape of the synaptic junctions, we did not observe any obvious differences in the ultrastructure of the presynaptic boutons contacting GC spines throughout the distinct maturation stages. All in all, these observations suggest that early proximal excitatory inputs on adult-generated GCs are formed within a few days of these cells arriving to the GC layer and that they then undergo a reorganization of PSDs (Figure 1D) during subsequent maturation stages.

### Functional characterization of early proximal glutamatergic inputs on new GCs

To investigate the functional properties of developing synapses in the GC layer, we monitored electrophysiological events using whole-cell patch-clamp recordings (Figure 2A). We first analyzed the profiles for both inward Na^+ ^currents and input membrane resistance (R_in_). Previous studies showed that inward Na^+ ^currents appeared mostly on class 5 newborn GCs; that is, one week after viral injection [8]. The presence of the apical dendrite was visualized both by GFP labeling and by loading cells with biocytin during patch-clamp recordings (Figure 1A). As expected, total dendritic length increased together with the increase in amplitude of the Na^+ ^current (see Additional file 1A). By contrast, an inverse correlation was found between the Na^+ ^current amplitude and R_in _(see Additional file 1B), known to decrease during neuronal maturation [8]. Therefore, the amplitude of the voltage-gated Na^+ ^current could be used as a reliable proxy of maturation stage (see Additional file 1C). Rather than categorizing recorded cells by the number of dpi (see Additional file 1D), we classified recorded individual GFP-positive GCs according to the combination of three parameters: 1) maximum Na^+ ^current amplitude, 2) dendrite length, and 3) R_in _values. Class 3 neurons, recorded at about 4 dpi, expressed a Na^+ ^current of <150 pA, dendrite length <100 μm and R_in _values >2.7 GΩ (see Additional file 1). Class 4 neurons, recorded at about 6 dpi, had a Na^+ ^current amplitude ranging from 200 to 800 pA, dendrite length of up to 280 μm and R_in _values of between 2.3 and 5 GΩ. Class 5 neurons, recorded at about 21 dpi, had a Na^+ ^current amplitude >800 pA, dendrite length of up to 310 μm and R_in _values <2.5 GΩ.

We then mapped connectivity for the recorded GCs using a minimal stimulation protocol delivered at proximal domains of GC dendrites. Previous studies suggested that excitatory glutamatergic inputs to the proximal dendrites of GCs originate from local collaterals of MC axons and centrifugal feedback projections from cortical regions [17]. However, these two inputs differ substantially in their location in the GC layer; centrifugal inputs remain deep in the GC layer [16,17]. Using this criterion, we focused our analysis on inputs showing the hallmarks of centrifugal feedback projections [17,26], bearing in mind that patch recordings are performed from the cell body whereas the synapses on the EM analysis originate from the dendritic spines some distance away on proximal dendrites. Starting with a low-intensity stimulation that did not evoke any excitatory postsynaptic currents (EPSCs), we then increased the stimulus strength gradually until a fast EPSC appeared. Only when a stimulating electrode was placed into the GC layer (Figure 2A) near the soma of the class 3 neurons did we observe EPSCs in an "all or nothing" manner (Figure 2B and Additional file 2). In our previous report [26], using a similar position for the stimulating electrode we observed EPSCs similar to those evoked by the axodendritic inputs in the proximal domain of the apical dendrite (Figure 2A). Stimulation near this recruitment threshold often failed to induce an EPSC. Slightly increasing the stimulation intensity beyond that threshold prevented the occurrence of failures, without affecting the amplitude of successful responses (see Additional file 2). In all cases, further small increments in stimulus intensity did not affect EPSC amplitude.

We characterized the development of synaptic inputs for the three classes of newly-formed neurons by using the voltage-clamp technique to measure isolated AMPAR-mediated EPSCs at a holding potential of -70 mV. After EPSCs were recorded at +40 mV with the same stimulus strength, we applied the AMPAR antagonist 2,3-dioxo-6-nitro-1,2,3,4-tetrahydrobenzo[f]quinoxaline-7-sulfonamide (NBQX) at 10 μmol/l. Finally, we confirmed, using the NMDAR antagonist D,L-2-amino-5-phosphonopentanoic acid (D,L-APV) at 100 μmol/l, that EPSCs were mediated by NMDAR. Gabazine (SR-95531) at 10 μmol/l was used to block GABA_A _receptor-mediated events and was present throughout all experiments. Minimal stimulation evoked both AMPAR-mediated and NMDAR-mediated EPSCs in newborn GCs in a success or failure manner (Figure 2B, C). After we subtracted mean failure responses from all responses (that is, success and failure) to prevent the obscuration by the stimulus artifact, we compared AMPAR-mediated and NMDAR-mediated EPSC amplitude among classes. NMDAR-mediated current amplitude gradually increased with more advanced maturation (class 3: 1.8 ± 0.6 pA; class 4: 5.0 ± 1.6 pA; class 5: 8.8 ± 1.4 pA) (Figure 2D). By contrast, AMPAR-mediated EPSC amplitude did not differ between class 3 and class 4 GCs (class 3: 4.6 ± 1.0 pA; class 4: 4.4 ± 0.9 pA), but was significantly increased in class 5 neurons (19.3 ± 7.6 pA; *p *< 0.05) (Figure 2E). These findings show that stimulation of OB slices, when delivered at minimal intensity, is a useful approach to study the maturation of a single presynaptic terminal contacting a maturing adult-generated GC.

To exclude the possibility that the abrupt increase in AMPAR-mediated EPSC amplitude at class 5 might reflect the transition from regimens in which Na^+ ^current is adequately clamped (class 3 and 4) to a regimen in which potentially it could not be clamped so well (class 5), we compared the amplitude of AMPAR-mediated outward EPSCs at the depolarized membrane potential. When these currents were derived from the subtraction of NMDAR-mediated components from EPSCs without NBQX, we obtained similar results (see Additional file 3). Taken together, these results suggest that NMDAR and AMPAR might have distinct roles during the maturation processes of adult-born GCs.

### Functional maturation of NMDAR- and AMPAR-mediated EPSCs

**Figure 3:**
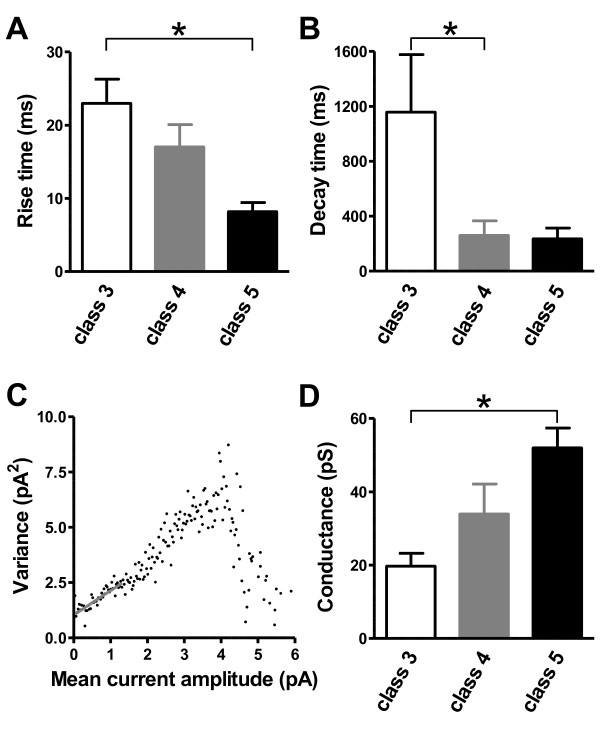
**Dynamic changes in NMDARs in maturing adult-generated GCs**. **(A) **The 10-90% rise time of NMDAR-mediated EPSCs became shorter with maturation (class 3 *vs *class 5; *P < 0.05) (class 3: five slices, five mice; class 4: nine slices, eight mice; class 5: four slices, four mice). **(B) **The decay time constant of NMDAR-mediated EPSCs was much lower in class 4 than in class 3 cells (class 3 versus class 4; *P < 0.05) (class 3: four slices, four mice; class 4: seven slices, six mice; class 5: four slices, four mice). (**C) **Peak-scaled variance-mean current for NMDAR-mediated EPSCs. The gray line was fitted linearly to the first 25% of the data points and used to estimate the weighted mean single open NMDAR channel conductance. **(D) **Fluctuation analysis showed an increase in NMDAR channel conductance during maturation (class 3 versus class 5; *P < 0.05) (class 3: four slices, four mice; class 4: eight slices, seven mice; class 5: four slices, four mice).

The kinetics of postsynaptic currents, including NMDAR-mediated and AMPAR-mediated events at various synapses, differ at different stages of neuronal maturation [31]. During the early stages of development, the predominant form of synaptic current is known to be mediated by NMDAR, which then declines rapidly with age and/or neuronal activity [32]. Thus, we examined in detail the kinetics of evoked EPSCs in developing GCs. The 10-90% rise times of NMDAR-mediated EPSCs gradually decreased during maturation (class 3: 23.0 ± 3.3 ms; class 4: 17.0 ± 3.0 ms; class 5: 8.2 ± 1.2 ms) (Figure 3A). The decay time constant of NMDAR-mediated EPSCs, which was characterized by a double exponential function, also significantly decreased over time (class 3: 1158 ± 419 ms; class 4: 260 ± 107 ms; class 5: 235 ± 77 ms) (Figure 3B). In line with these results, developmental switches in NMDAR subunits are known to shorten NMDAR-mediated EPSC durations [33].

The conductance of ionotropic glutamate receptors depends on subunit composition [34]. We therefore used non-stationary fluctuation analysis to estimate single-channel conductance [35,36]. The relationship between the variance and mean amplitude of NMDAR-mediated current was found to be skewed (Figure 3C). We fitted the initial slope to estimate the mean single-channel current underlying NMDAR-mediated EPSCs, and then calculated the single channel conductance. Results showed a gradual increase in NMDAR single-channel conductance over time (class 3: 19.7 ± 3.5 pS; class 4: 33.9 ± 8.2 pS; class 5: 52.0 ± 5.4 pS) (Figure 3D).

**Figure 4:**
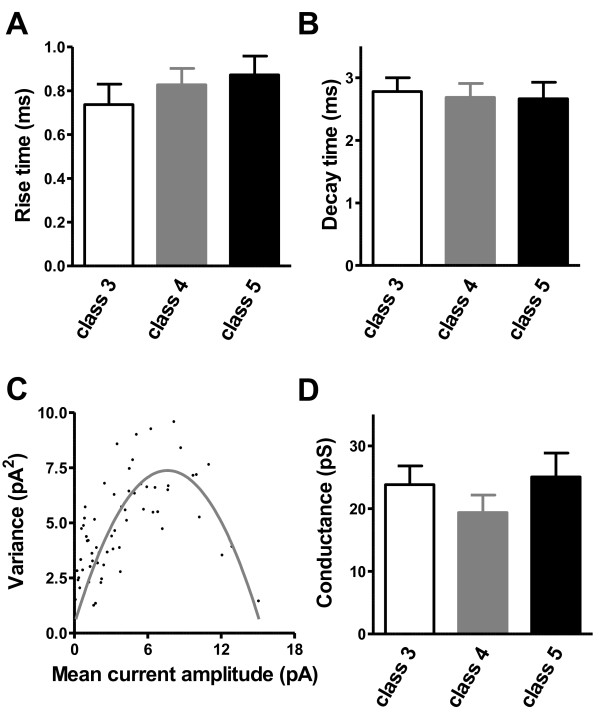
**AMPAR-mediated EPSCs during maturation of adult-generated GCs**. **(A) **The 10-90% rise time of AMPAR-mediated EPSCs did not differ between different stages of maturation (class 3: eleven slices, eleven mice; class 4: sixteen slices, fourteen mice; class 5: nine slices, seven mice). **(B) **Similar decay time constants were observed for the different classes of developing adult newborn GCs (class 3: seven slices, seven mice; class 4: ten slices, nine mice; class 5: nine slices, seven mice). **(C) **Variance-mean current relationship from peak-scaled non-stationary fluctuation analysis. Gray line shows a parabolic relationship. **(D) **Estimated single open channel conductance for AMPARs. Fluctuation analysis did not show any significant change in AMPAR channel conductance during maturation (class 3: seven slices, seven mice; class 4: twelve slices, eleven mice; class 5: nine slices, seven mice).

By contrast, the properties of AMPAR-mediated EPSCs remained unchanged over the same time window (Figure 4). AMPAR-mediated EPSCs showed a similar 10-90% rise time for the different stages of maturation (class 3: 0.7 ± 0.1 ms; class 4: 0.8 ± 0.1 ms; class 5: 0.9 ± 0.1 ms) (Figure 4A) and similar decay time constants (class 3: 2.8 ± 0.2 ms; class 4: 2.7 ± 0.2 ms; class 5: 2.7 ± 0.3 ms) (Figure 4B). Non-stationary fluctuation analysis showed a parabolic relationship between the variance and the mean EPSC amplitude (Figure 4C). The mean single-channel current was thus derived from the parabolic function. Once again, single-channel conductance did not significantly differ among maturation stages (class 3: 23.9 ± 3.0 pS; class 4: 19.4 ± 2.8 pS; class 5: 25.1 ± 3.8 pS) (Figure 4D). These findings suggest that NMDARs, but not AMPARs, undergo a functional rearrangement during development, including, for example, a change in subunit composition.

### Mechanism of transmitter release at newly-formed synapses

**Figure 5:**
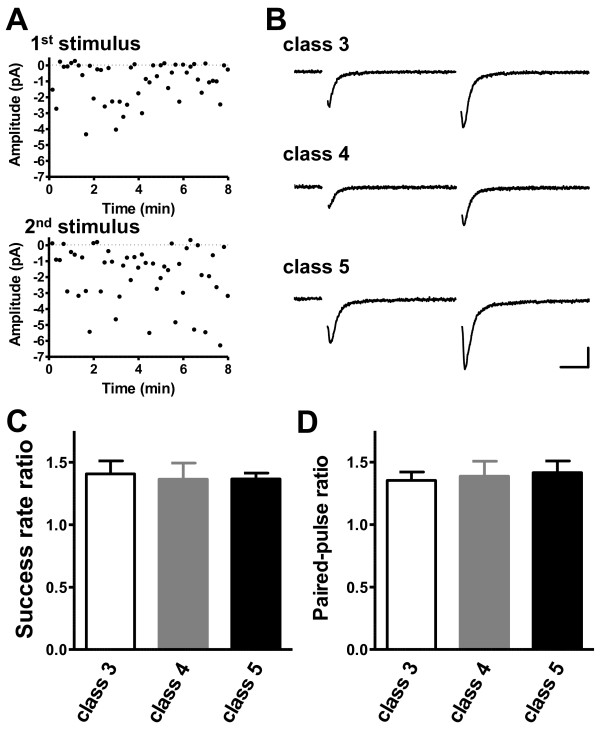
**Short-term plasticity of developing adult-generated GCs**. **(A) **AMPAR-mediated EPSC amplitude plotted against time for responses to first (top) and second (bottom) stimuli during paired-pulse stimulation. **(B) **Paired-pulse ratios determined for successful responses (class 3: six slices, six mice; class 4: eleven slices, nine mice; class 5: eight slices, seven mice). **(C) **Mean responses (successes and failures) to paired-pulse stimulation. Scale bar = 10 ms and 5 pA. (D) Paired-pulse ratios for mean AMPAR-mediated EPSC amplitude (successes and failures) (class 3: six slices, six mice; class 4: eleven slices, nine mice; class 5: eight slices, seven mice).

Functional changes in proximal synapses on new GCs may be simultaneously regulated at postsynaptic and presynaptic sites. We examined presynaptic features using a paired-pulse protocol (interstimulus interval 50 ms) (Figure 5A) previously used to measure the efficacy of neurotransmitter release from the presynaptic terminal [37]. The ratio of the success rate of the second response to the success rate of the first response was >1 and remained constant for all neuronal classes (class 3: 1.41 ± 0.10; class 4: 1.36 ± 0.13; class 5: 1.37 ± 0.04) (Figure 5B). We also compared the ratio of the average amplitude of AMPAR-mediated EPSCs (all successes and failures) for the first stimulus to that of the second for developing GCs (Figure 5C). The degree of facilitation in response to paired-pulse stimuli was similar for all classes (class 3: 1.35 ± 0.07; class 4: 1.39 ± 0.12; class 5: 1.42 ± 0.09) (Figure 5D), which suggested that maturation of new GCs was not linked to a change in the probability of glutamate release. Thus, the rapid increase in the NMDAR/AMPAR ratio over time results primarily from postsynaptic changes.

**Figure 6:**
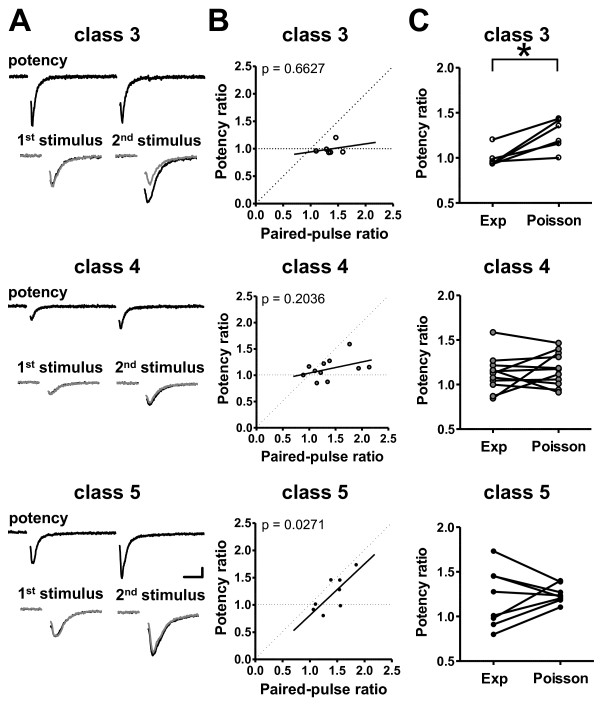
**Changes in release sites contacting developing adult-generated GCs**. **(A) **Potency values obtained for the same neuron as in Figure 5C. Upper trace for each class: mean potency for first and second stimuli (for successful responses only). Bottom trace for each class: AMPAR-mediated EPSC amplitude in response to the first stimulation was scaled (black) to potency values obtained for AMPAR-mediated EPSC amplitude (gray). This scaling shows the difference between AMPAR-mediated EPSC amplitude and potency values for AMPAR-mediated EPSC amplitude after the second stimulation. Note that class 3 cells have the greatest difference. Scale bar = 10 ms, 5 pA. (B) Potency ratio (for successful responses only) as a function of AMPAR-mediated EPSC amplitude ratio (successes and failures). Horizontal and diagonal dashed lines indicate an increase in the probability of release at single or multiple release sites, respectively. The linear regression line fitted to all data points for each class is shown in black. *P *< 0.05 indicates significant deviation of the slope from zero. **(C) **Comparison between observed (Exp) and predicted (Poisson) potency ratios. Predicted values were obtained using a Poisson model. Note that a significant difference was only observed in class 3 cells (*P < 0.05). Data are from the same cells as in Figure 6B.

The combination of minimal and paired-pulse stimulation techniques allowed analysis of the evoked EPSCs for which transmitter release was detected in response to both the first and second stimulation (Figure 6A). We measured mean amplitudes for the first and second responses, considered as the 'potency' of each pulse. Only class 3 GCs had similar potency values for both responses. If one proximal synapse has only one functional release site, zero or one quantum can be released. Thus, if both the first and second pulses generated successful responses, releasing one quantum, there would be no difference in the size of these responses; that is, the potency ratio (second pulse to first pulse) would be 1. However, if multiple release sites contribute to the synaptic response, the second pulse may induce the simultaneous release of multiple transmitter quanta, generating a larger synaptic response for the second pulse than for the first. Figure 6B shows the potency ratio plotted as a function of the ratio of the mean amplitude of evoked EPSCs (successes and failures). Most data from class 5 cells, but not from class 3 or 4 cells, could not be expressed as a horizontal line (potency ratio = 1). When a linear regression line was fitted to all the data, the slope increased significantly during maturation (class 3: 0.15 ± 0.31; class 4: 0.21 ± 0.15; class 5: 0.94 ± 0.32). The slope was significantly non-zero only for class 5 GCs (class 3: *P *= 0.66; class 4: *P *= 0.20; class 5: *P *= 0.03). Thus, these data suggest that more than one single release site accounts for the synaptic responses recorded in class 5 cells. Changes in potency could be simulated by success rates of the experimental data using a simple Poisson release model (Figure 6C). This model assumes activation of multiple functional release sites with low release probability [38-40]. The predicted potency ratio was significantly greater than the measured ratio for class 3 GCs only (*p *= 0.01) and this difference between predicted and measured potency ratios disappeared with maturation (class 4: *P *= 0.43; class 5: *P *= 0.64). These results suggest that the number of functional release sites at proximal synapses may increase from class 3 to class 5.

### Properties of proximal synapses on mature GCs

**Figure 7:**
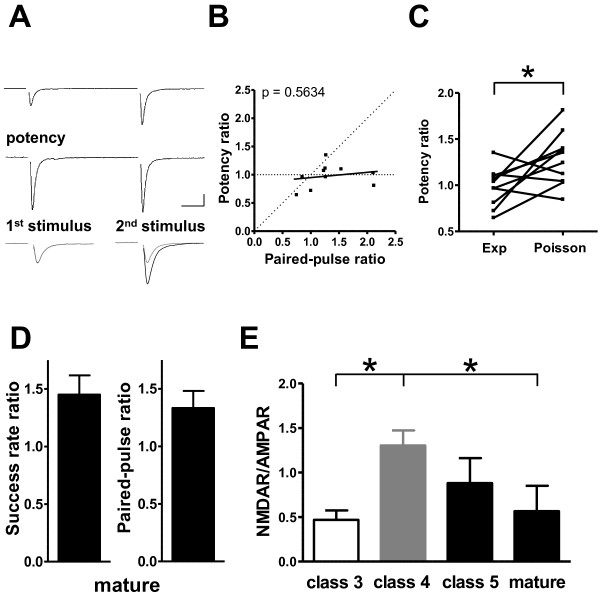
**Presynaptic properties of synapses contacting developing and mature GCs**. **(A) **Typical responses to paired-pulse stimulation delivered at the proximal site. Top: Mean responses for the whole dataset (all successes and failures). Middle: Mean potency values for responses to the first and second rounds of stimulation (successful responses only). Bottom: AMPAR-mediated EPSC amplitude and potency. AMPAR-mediated EPSC amplitude for responses to the second round of stimulation, after AMPAR-mediated EPSCs amplitude to first stimulation was scaled (black) to potency for AMPAR-mediated EPSCs amplitude (gray). Scale bar = 10 ms and 10 pA. (B) Paired-pulse ratios for successful responses (left) (ten slices, six mice) and AMPAR-mediated EPSC amplitude (right), recorded from mature GCs (10 slices, six mice). **(C) **Left: Potency ratio (successful responses only) plotted as a function of AMPAR-mediated EPSCs amplitude ratio (successes and failures) recorded from mature GCs. Most points fell on or near the horizontal dashed line. The linear regression line fitted to all data points is shown in black. *P *< 0.05 indicates significant deviation of the slope from zero. Right: Comparison between observed (exp) Yes and predicted (Poisson) potency ratios. Predicted values were obtained using a Poisson model. Data were for mature GCs. **(D) **Ratio of AMPAR-mediated to NMDAR-mediated EPSC amplitude increased during the early developmental stage (class 3 versus class 4, class 4 versus mature; *P < 0.05) (class 3: six slices, six mice; class 4: nine slices, eight mice; class 5: four slices, four mice; mature: six slices, five mice).

We also examined the electrophysiological properties of class 5 cells at later time points after viral injection. At about 14 dpi, newborn class 5 GCs exhibit long-term potentiation (LTP) at proximal glutamatergic synapses, but this plasticity disappears over time [26]. Thus, the properties of the input synapses on fully mature GCs may differ from those of synapses on class 5 cells. To examine this possibility, two consecutive stimuli of minimal intensity were delivered to evoke neurotransmitter release from presynaptic terminals contacting mature non-GFP-labeled GCs (Figure 7A). The success rate of the second response exceeded that of the first (a ratio of 1.45 ± 0.17) (Figure 7B). Paired-pulse stimulation also resulted in the facilitation of the mean AMPAR-mediated current amplitude in pre-existing mature GCs (a ratio of 1.33 ± 0.15) (Figure 7A). Neither the success rate ratio nor the paired-pulse ratio differed between developing and mature GCs. The predicted potency ratio was significantly greater than the observed ratio in mature GCs (Figure 7C). Consistent with this result, the linear regression line that fitted data obtained from mature GCs was almost parallel to the horizontal line (slope 0.09 ± 0.15) (Figure 7C). The predicted potency ratio by a simple Poisson release model was also significantly greater than the measured ratio for mature GCs (*P *< 0.03). Similar to the findings for class 3 cells, these results indicate that mature GCs receive proximal synapses characterized by a single functional release site.

Although the amplitudes of AMPAR-mediated and NMDAR-mediated EPSCs recorded from mature GCs were significantly higher than those from class 3 GCs (AMPAR-mediated events 26.7 ± 8.2 pA; NMDAR-mediated events 11.8 ± 5.1 pA), the NMDAR/AMPAR ratios for mature and class 3 GCs were indistinguishable (Figure 7D). This ratio was maximal for class 4 and class 5 neurons (class 3: 0.47 ± 0.11; class 4: 1.30 ± 0.17; class 5: 0.88 ± 0.28; mature: 0.57 ± 0.28). These findings suggest that AMPARs are functionally dominant at proximal synapses in both class 3 neurons and mature GCs.

## Discussion

**Figure 8:**
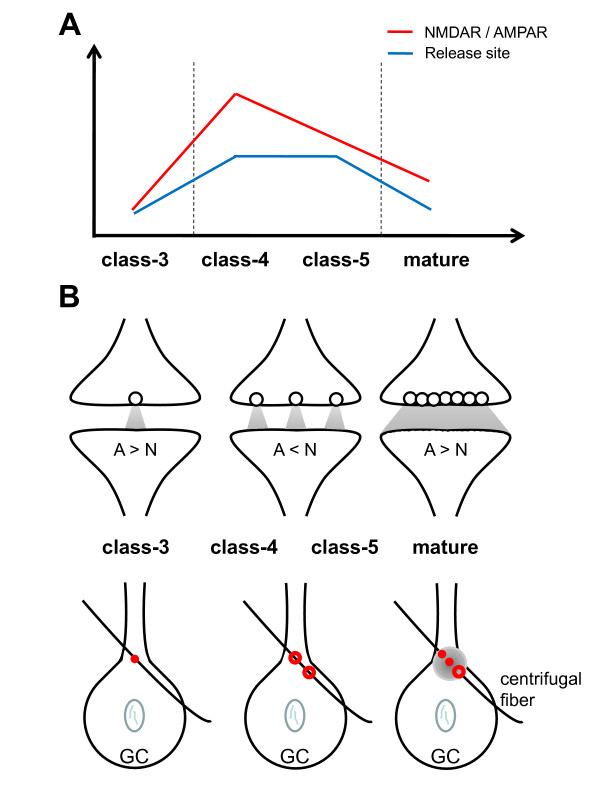
**Schematic diagram of an axodendritic synapse for each class of GC**. **(A) **Time-course analysis of two elements during the maturation of the first glutamatergic contact onto newborn GCs. The NMDAR/AMPAR ratios are shown in red. Functional release sites (blue) are indicated as either single or multiple. Both class 4 and class 5 neurons showed a high NMDAR/AMPAR ratio with multiple release sites. **(B) **Developmental changes in a single axodendritic synapse. Top: Transmitter vesicular events (circle) per action potential gradually changed from single to multiple events. Each event occurred independently, but seemed to become integrated together in mature GCs (gray). Bottom: Differences observed in the number of axodendritic synapses. During maturation, the number of synapses (red circles) impinging onto newborn GCs increased in parallel with the appearance of perforated synapse (red doughnut-like shape). At the later stages of maturation, perforated synapses then divided, with a spillover of transmitter occurring at adjacent synapses (gray).

This study shows that early functional glutamatergic inputs contact the developing adult-born GCs at proximal sites, well before glutamatergic synapses appear at the distal domain, consistent with previous studies [11,13,32]. Our EM analysis of the GC layer, together with other physiological studies [17,25,26], identifies these proximal inputs as axodendritic synapses originating from a variety of brain regions. These include projections from pyramidal cells in secondary olfactory cortical areas, frontal cortex and hippocampal structures [22,41,42]. Our approach demonstrates, for the first time, a functional maturation pattern of the synaptic elements at these glutamatergic inputs (Figure 8A). The NMDAR/AMPAR ratio reached maximum in class 4 cells, with lower values observed in more immature or mature GCs. This time window precisely corresponds to the critical period during which the survival of newborn GCs is highly sensitive to sensory experience [43] and needs functional NMDARs [44]. Similarly, multiple functional release sites were detected only in class 4 and 5 cells during the same time window. At the end of the critical period, the glutamatergic terminals revert slowly to bearing only a single release site. This study emphasizes the precise orchestration for the maturation of presynaptic and postsynaptic elements at the synaptic proximal inputs onto adult-born GCs, in contrast to the situation for neonatal GCs, where dendrodendritic synapses first appear before axodendritic synapses [27,28].

### Formation of the first glutamatergic contacts

Class 1 and 2 cells are migratory neurons [29], which express AMPAR before reaching their final position in the bulb [8,45]. AMPAR-mediated current recorded from these migrating cells showed an almost linear current-voltage relationship, indicative of Ca^2+^-impermeable AMPARs [8]. By contrast, AMPAR-mediated currents at the proximal synapses of class 3 cells showed inward rectification (see Additional file 4) indicative of the presence of Ca^2+^-permeable AMPAR channels, as it has been recently demonstrated using flash photolysis of caged glutamate [46]. Supporting this assumption is the large single channel conductance for AMPAR estimated from our non-stationary noise analysis (Figure 4D), as previously reported [47]. Intracellular Ca^2+ ^is thought to play an important role in regulating the migration and maturation of newborn neurons [45,48]. Further experiments will determine whether this Ca^2+ ^permeability mediates a stop signal that halts the radial migration of new GCs, as demonstrated for migrating neurons of the embryonic cortex [49].

Class 3 GCs that have just reached the OB show limited dendritic arborization (< 100 μm) and a virtual absence of spines; however, we detected early functional glutamatergic inputs at this time point (Figure 2). Meanwhile, newborn neurons in the hippocampus receive their first glutamatergic synapses about two weeks after developing in the GC layer [50]. Further studies should investigate whether the difference in the onset of glutamatergic inputs might support differences in new neuron turnover between the OB and hippocampus, as previously reported [51].

We found that a single axon terminal formed synapses onto the dendrites of both GFP-positive and GFP-negative GCs (see Additional file 5). This suggests that immature and mature synapses share the same presynaptic input, assuming that GFP-negative cells represent mature pre-existing GCs. Notably, the distance between synapses in immature and pre-existing GC dendrites can be as small as < 1 μm (see Additional file 5). Such proximity suggests that new GCs may integrate into the mature circuit using pre-existing terminals, as previously described for new neurons of the adult dentate gyrus [52]. Upon arrival of a new GC, glutamate release from the pre-existing terminals may trigger the extension of dendritic filopodia, resulting in the formation of postsynaptic specializations. At the same time, directed recruitment of mobile vesicles to nascent active zones in pre-existing terminals [53] may lead to the formation of additional presynaptic release sites (Figure 8B, bottom). One potential benefit of such a coordinated mechanism would be the ability to rapidly initiate the formation of new synapses.

### Maturation of glutamatergic synapses at the postsynaptic site

When a presynaptic fiber contacts the proximal dendrites of class 3 neurons, mobile transport packets, containing presynaptic and postsynaptic proteins, are immediately recruited for synaptic maturation [53]. We observed that the amplitude of EPSCs mediated only by NMDAR was higher in class 3 than in class 4 cells. Furthermore, NMDARs may also play an active role in keeping immature synapses free from AMPARs until an appropriate trigger signals the recruitment of this receptor, as described in developing circuits [54]. Thus, the incorporation of NMDARs may precede the insertion of AMPARs during the early maturation stages in adult newborn GCs.

We also found a shorter duration of NMDAR-mediated currents in class 4 neurons than in class 3 neurons. Increasing evidence suggests an acceleration of NMDAR-mediated events during neuronal maturation, reflecting a switch in subunit composition [55]. NMDARs consist of two obligate NR1 subunits and two NR2A-D or NR3A-B subunits, the recruitment of which depends on spatial and developmental signals [56]. Our findings indicate that NMDAR channel conductance increased from low to high conductance during the maturation process, possibly because the NMDAR-mediated currents recorded in class 3 cells were presumably mediated by NR2D-containing receptors, known to support low conductance states [57]. Reconstructed systems have shown that NR2D-containing receptors mediate currents with a long decay-time constant [58], consistent with the prolonged decay-time constant observed in class 3 cells. Additionally, morphological analyses have shown that the NR2 D subunit is mainly located at extrasynaptic sites [59]. The slow rise time of NMDAR-mediated currents recorded from the centrifugal fibers of class 3 cells could result from the activation of extrasynaptic NR2D-containing receptors. Then, during maturation of newborn cells from class 3 to class 4, high-conductance NMDARs may be recruited from extrasynaptic to synaptic sites. This scenario would be consistent with the shortened rise time of NMDAR-mediated currents observed at the later maturation stages; however, pharmacological experiments should be conducted to directly test this possibility.

During development, glutamatergic synaptic currents undergo a characteristic pattern of maturation involving changes in the kinetics of NMDAR-mediated currents. The formation of 'silent' synapses, which display only NMDAR-mediated currents and which are later made functional through recruitment of AMPARs, is also a possibility. These changes in glutamatergic synapses during maturation have implications for the early development of neural networks [60] and for the mechanisms underlying the LTP of synaptic efficacy [61]. In our study, we detected the highest NMDAR/AMPAR ratio in class 4 cells, even though axodendritic synapses are not 'silent' at the stage of class 3. This high NMDAR/AMPAR ratio declined with neuronal age as a result of AMPAR recruitment. This observation suggests that the development of axodendritic synapses after the class 4 stage (that is, from 6 dpi) somewhat recapitulates the mechanism of maturation described during brain development.

### Changes in synapse ultrastructure on developing GCs

Our study found changes in functional release sites during the maturation process. Minimal stimulation experiments revealed the presence of a single functional release site in class 3 GCs and multiple functional release sites in both class 4 and class 5 GCs. Previous studies have shown that multiple vesicles might be released at each synaptic contact for each action potential fired [62,63]. These changes could reflect a maturation of individual synapses, involving developmental switching from single to multiple release sites (Figure 8B, top) [62,63]. Indeed, previous ultrastructural analyses have demonstrated that developing (class 3) neurons establish immature synapses characterized by small cluster of vesicles (that is, a group of >4 vesicles) and faint membrane specializations [11], whereas mature synapses contain large cluster of vesicles and substantial asymmetric membrane thickening [11] (and this study).

Each neurotransmitter release site in the mature brain functions in a binary mode, releasing one or zero quantum of neurotransmitter for each action potential [38,64]. Therefore, many excitatory synapses in the mammalian brain can release a maximum of the contents of one vesicle [65]. Alternatively, the changes described here may reflect changes in the number of synaptic appositions contacting a given GC (Figure 8B, bottom). A single synapse may first develop into a more complex contact (involving the presence of perforations) and then split into two synapses, still connected to the same single axon. Interestingly, LTP has been reported to initiate the perforation of spines, leading to the appearance of multiple spine boutons [66]. Because LTP has been observed at the proximal synapses of GCs [25,26], it is possible that the changes in the number of synaptic contacts we found might reflect some form of synaptic plasticity.

At later stages of maturation, we found that GCs revert to a single functional release site. The paired-pulse ratio was smaller after supraminimal stimulation than after minimal stimulation in mature, but not in class 5 GCs ([26]) (Figure 7B). This could arise through presynaptic events, such as successful release at most release sites triggered by the first supraminimal pulse, or through postsynaptic events, such as saturation of receptors after multivesicular release [67]. Two possible models may thus be considered for the axodendritic synapse of mature GCs: individual boutons displaying multiple dense release sites (Figure 8B, top) or synaptic connections with multiple release sites in close proximity (Figure 8B, bottom). In both cases, multivesicular events occur in synchrony at the axodendritic synapse of more mature GCs.

## Conclusion

In this study we found that, within a few days after having reached the OB, the first glutamatergic synapses underwent a dynamic developmental process. This proximal excitatory input is subjected to both physiological and morphological modifications before reciprocal dendrodendritic synapses start to operate at distal sites. Our results provide a conceptual framework for understanding mechanisms underlying the precise control of neural wiring through presynaptic and postsynaptic events during adult neurogenesis. It is remarkable that both basic physiological properties (this study) and the synaptic plasticity [26] were found to be continuously changing over time, even when the ultrastructure of newly-formed synapses became similar to that of mature counterparts. This finding contrasts with the observation that GCs in the adult dentate gyrus continue to change physiologically and morphologically after the formation of their first glutamatergic synapses [50,68,69]. We indicate here that the influence of adult OB neurogenesis may be gradually tuned by early glutamatergic synaptic transmission, depending on the activity of cortical structures downstream from the OB. Elucidating the origin of the glutamatergic centrifugal fibers impinging so early onto newborn GCs, and the molecular mechanisms that shape the integration of new neurons, has important implications both for understanding how OB circuits are refined by experience and for the successful use of stem cell-based replacement therapies in brain repair.

## Methods

### Mice

C57BL/6J male mice about eight weeks old (Janvier, France) were used for all experiments. All procedures were carried out in accordance with the EU Charter of Fundamental Rights (2000/C 364/01) and the European Communities Council Directive of 24 November 1986 (86/609/EEC), and were reviewed and approved by our institutional animal welfare committee.

### Lentiviral vectors

For cytosolic GFP labeling, a custom-built lentivirus containing the GFP gene under the control of the PGK promoter was used to transduce adult-born neurons as previously described [12]. Viruses (2.2 × 10^10 ^transducing units (TU)/ml) were stored at -80°C. Immediately before injection, the lentiviral vectors were diluted in phosphate-buffered saline (PBS) to a final concentration of 15 ng of p24 protein per microliter.

### Stereotaxic surgery

For stereotaxic injections of lentiviral vectors, adult mice about six weeks old were anesthetized with a mixture of ketamine (Imalgene^®^, Merial, Lyon, France) (1.5% in PBS) and xylazine (Rompun^®^, Bayer Health Care, Puteaux France) (0.05%; 250 μl per mouse). Mice were mounted in a Kopf stereotaxic apparatus, and small craniotomies were created above the injection sites. Virus was injected into the rostral migratory stream (RMS) at the following co-ordinates in each hemisphere: anteroposterior +3.3 mm from bregma; mediolateral ± 0.82 mm from bregma; and dorsoventral -2.85 mm from pial surface. Injected mice were housed individually until used for electrophysiological experiments. Injections of the viral vector into the RMS specifically labeled adult-born migrating neuroblasts, with OB slices showing GFP expression in newborn interneurons only. We did not detect any diffusion from the injection site resulting in transduction of the resident OB cell population during the course of these experiments. This approach has been successfully used previously to characterize the morphological maturation of adult-born periglomerular cells [10].

### Slice preparation

Mice were deeply anesthetized with isoflurane (Mundipharma, Issy-les-Moulineaux, France) and then rapidly decapitated. Horizontal slices from the OB and frontal cortices were obtained after decapitation and brain removal. After cutting, slices (300 μm) were placed in oxygenated artificial cerebrospinal fluid (ACSF) at 35°C for 30 min. Slices were then kept in bubbled ACSF at room temperature. ACSF contained 124 mmol/l NaCl, 3 mmol/l KCl, 1.3 mmol/l MgSO_4_, 26 mmol/l NaHCO_3_, 1.25 mmol/l NaHPO_4_, 20 mmol/l glucose and 2 mmol/l CaCl (all chemicals from Sigma-Aldrich, Saint-Quentin, France).

### Whole-cell patch-clamp recordings

Individual slices were placed in a submerged recording chamber and were continuously perfused with ACSF (1.5 ml/min) at room temperature. Whole-cell voltage-clamp recordings from GCs in the GC layer were obtained using a ×40 water-immersion objective, a halogen light source, differential interference contrast filters (all Olympus, Rungis, France), a charge-coupled device (CCD) camera (C7500; Hamamatsu, Japan) and an amplifier (EPC9/2; Heka Instrument, Port Washington, USA). Patch electrodes (6-10 MΩ) were filled with an internal solution (126 mmol/l Cs gluconate, 6 mmol/l CsCl, 2 mmol/l NaCl, 10 mmol/l Na-HEPES, 10 mmol/l D-glucose, 0.2 mmol/l Cs-EGTA, 0.3 mmol/l GTP, 2 mmol/l Mg-ATP, 0.2 mmol/l cAMP and 0.15% biocytin at pH 7.3 and 290-300 mOsm). Liquid junction potentials (9 mV) were compensated. Maximum Na^+ ^current amplitude, measured by increasing the magnitude of depolarizing pulse by 10 mV increments, was mainly used to classify newborn GCs into three classes. Individual cell class was established from recording GFP-positive cells within the following range of days after virus injection: class 3 a 3 to 6 dpi; class 4 at 4 to 10 dpi and class 5 at 6 to 44 dpi (see Additional file 1D).

The stimulating electrode was placed near a recorded GC (Figure 2A) [26]. Response amplitude was adjusted as required for a minimal stimulation protocol to stimulate only one axon that was directly presynaptic to the recorded GC. Stimulus intensity was gradually increased from a low level until an EPSC suddenly appeared in an all or nothing manner (see Additional file 2). Single axons were stimulated at 0.1 Hz, after checking that the mean EPSC amplitude for successful responses was not affected by small changes in stimulus intensity. Recordings were filtered at 10 kHz (Filter 1) and 2.9 kHz (Filter 2), digitized, and sampled at intervals of 20 to 450 μs (2.2 - 50 kHz) according to the requirements of individual protocols [12]. Series resistance (< 30 MΩ) was monitored for stability throughout the recordings and if it had a change of >20%, the data were discarded. All experiments were performed in the presence of SR-95531 (gabazine) to block GABA_A _receptor-mediated currents.

Mean values from failure traces were subtracted from those of all responses (successes and failures) to measure response amplitude, 10-90% rise times, and decay time constants. Decay time was calculated by the formula:

$$I\left(t\right)=I_{f}\exp\left(-t/t_{f}\right)+I_{s}\exp\left(-t/t_{s}\right),$$

where *I_f _*and *I*_*s *_are the peak amplitudes of fast and slow components, respectively, and *t_f _*and *t_s _*are the corresponding time constants. For comparison, weighted mean decay time constants were calculated by:

$$t_{w}=t_{f}\left[I_{f}/\left(I_{f}+I_{s}\right)\right]+t_{s},\left[I_{s}/\left(I_{f}+I_{s}\right)\right]$$

To estimate single channel conductance, the mean current waveform was scaled to the peak of each synaptic current. The variance of each synaptic current around the scaled mean curve was then calculated. These variances were divided into 200 equally sized bins based on amplitude, and plotted against the mean current value within each scaled mean current bin. All data points were fitted by:

$$\sigma^{2}-\sigma_{basal}{}^{2}=i*I-I^{2}/N,$$

Where *i *is the current carried by a single open channel, and *N *is the number of open channels. NMDAR-mediated EPSCs showed a skewed variance versus mean relationship. The single-channel conductance for NMDAR-mediated currents was estimated by fitting the initial slope of the relationship (Figure 3C). For AMPAR-mediated EPSCs, the variance versus mean relationship was parabolic. Single-channel conductance for AMPAR-mediated currents was therefore estimated using *i*, and the difference between holding potential and reversal potential (Figure 4C).

For paired-pulse stimulation, two consecutive stimuli were delivered with an inter-pulse interval of 50 ms. The AMPAR-mediated EPSC amplitude was taken as the mean for all successes and failures (Figure 5; Figure 7), whereas potency was calculated from the mean successful responses only (Figure 6; Figure 7). The paired-pulse ratio was calculated as the mean EPSC response to the second pulse divided by mean EPSC response to first pulse (Figure 5; Figure 6; Figure 7). Simulated data were generated using a Poisson model: potency ratio:

$$(EPSC_{2}/EPSC_{1})=\left(\ln\left[1-SR_{2}\right]/\ln\left[1-SR_{1}\right]\right)*\left(SR_{1}/SR_{2}\right).$$

One or two slices were prepared from each virus-injected mouse and individual data were obtained from individual slices. Results are reported as mean ± SEM. A paired *t*-test was used for statistical analysis (Figure 6C; Figure 7C; Additional file 4). Kruskal-Wallis test followed by the Dunn multiple comparison test were also used to evaluate the data.

### Immunohistochemistry

Tissue slices containing biocytin-loaded cells were fixed in 4% paraformaldehyde at 4°C overnight. Slices were then washed three times in 0.1 mol/l phosphate buffer pH 7.4, without resectioning, and incubated with PBS containing Alexa 546 conjugated-Streptavidin (Molecular Probes, http://www.probes.invitrogen.com) and 0.25% Triton-X for 2 hours at room temperature. After washing three times in phosphate buffer, slices were observed under a confocal microscope (TCS SP5; Leica Wetzlar, Germany).

### Pre-embedding EM immunocytochemistry

Mice (7 dpi and 21 dpi) were anesthetized with pentobarbital and perfused with 2% PFA and 0.1% glutaraldehyde in sodium acetate buffer pH 6 for 2 minutes followed by 1 hour perfusion with 2% PFA and 0.1% glutaraldehyde in 0.1 mol/l borate buffer pH 9. Brains were post-fixed for 4 h with OBs cut into 70 μm coronal sections on a vibrating blade microtome (VT1200; Leica). The sections were cryoprotected with 30% sucrose and freeze-thawed three times to enhance antibody penetration. Sections were then processed for immunoperoxidase using primary antibodies against GFP (1:20,000; Chemicon International, http://www.chemicon.com). The peroxidase reaction product was silver-intensified and gold-toned as described previously [70]. Serial thin sections were collected on copper slot grids and examined under a transmission electron microscope (JEM-1010; Jeol, Tokyo Japan) equipped with a side-mounted CCD camera (Mega View III, Olympus Soft Imaging Systems, Brandenburg Germany) at a magnification of 30,000. Synaptic contacts were analyzed in images taken from at least five consecutive sections. Glutamatergic (type 1) synapses were recognized by the presence of vesicles in the presynaptic terminal and by a prominent postsynaptic density (asymmetric junctions). Three-dimensional reconstructions were generated with the software Reconstruct (J.C. Fiala, Biology Department, Boston University, Boston, USA), using digital images acquired from each serial section.

## Competing interests

The authors declare that they have no competing interests.

## Authors' contributions

HK contributed to the concept, designed, performed the electrophysiological experiments and analyzed the data. MP and AN performed morphological and electrophysiological experiments, respectively. KM prepared lentiviruses. HK, MS-P and P-ML. wrote the manuscript.

## Supplementary Material

Additional file 1 **Supplementary Figure 1: Classification of developing adult-generated granule cells (GCs)**. **(A) **Relationship between length of apical dendrite and maximal Na^+ ^current evoked by a depolarizing step pulse. Adult-born GCs were classified according to Na^+ ^current amplitude, with white, gray and black circles representing classes 3, 4 and 5, respectively. Apical dendrites elongated during maturation. **(B) **Inverse correlation between the input membrane resistance (R_in_) and maximum Na^+ ^current amplitude evoked by a depolarizing step pulse. **(C) **Plots of maximum Na^+ ^current amplitude evoked by a depolarizing voltage step versus days post-injection (dpi) of virus. **(D) **Records from various newborn GCs after viral injection. Note the absence of clear boundaries among classes. Click here for file

Additional file 2 **Supplementary Figure 2: Proximal synaptic responses evoked by minimal stimulation**. Gradually increasing stimulus intensity abruptly evoked events in an all-or-none manner. Open and closed circles indicate response success and failure, respectively. Unitary response amplitude was confirmed to be constant by a small increase in the stimulus intensity. Click here for file

Additional file 3 **Supplementary Figure 3: Developmental change of 3-hydroxy-5-methyl-4-isoxazolepropionic acid receptor (AMPAR)-mediated outward currents at depolarized membrane potentials**. (A) Typical traces. After AMPAR-mediated and *N*-methyl D-aspartate receptor (NMDAR)-mediated currents were recorded at the holding potential of +40 mV, 2,3-dioxo-6-nitro-1,2,3,4-tetrahydrobenzo[f]quinoxaline-7-sulfonamide (NBQX) was applied to obtain NMDAR-mediated excitatory postsynaptic currents (EPSCs). Traces were derived from the subtraction of NMDAR-mediated EPSCs from AMPAR-mediated and NMDAR-mediated currents. Scale bar = 1 ms and 1 pA. **(B) **The amplitude of AMPAR-mediated EPSCs was much higher in class 5 than in class 3 cells (class 3: 1.43 ± 0.43; class 4: 1.52 ± 0.34; class 5: 7.69 ± 3.15) (class 3 versus class 5, *P < 0.05) (class 3: six slices, six mice; class 4: eight slices, eight mice; class 5: four slices, four mice). Click here for file

Additional file 4 **Supplementary Figure 4: Ca^2+^-permeable AMPARs in class 3 GCs**. **(A) **(Black) AMPAR-mediated EPSCs at the holding potential of -70 mV (red). This trace shows predicted data, obtained by inversion of the trace shown in black and multiplication by four-sevenths. **(B) **(Green) AMPAR-mediated and NMDAR-mediated EPSCs at the holding potential of +40 mV. (Pink) NMDAR-mediated EPSCs at the same holding potential. This current was recorded in the presence of NBQX. (Blue) AMPAR-mediated current at the holding potential of +40 mV. This experimental trace was generated by subtracting the NMDAR-mediated component from both receptor-mediated currents (Green minus pink). **(C) **Comparison between the predicted and experimental traces. **(A-C) **Scale bar = 1 ms and 2 pA. **(D) **The maximum amplitude of the trace obtained from dividing experimental values by predicted values. If AMPAR-mediated EPSCs in class 3 GCs are mediated by Ca^2+^-impermeable AMPARs, this ratio would be equal to 1 (dashed line), given the linear current-voltage relationship of Ca^2+^-impermeable AMPAR. The experimental values obtained were significantly lower than the predicted values (class 3: six slices; *P < 0.05). Click here for file

Additional file 5 **Supplementary Figure 5: Axon terminals make contacts with both adult-born and pre-existing GCs**. A large axon terminal (Ax: red) makes two synapses with a GFP-positive dendrite (GC at 7 days post-injection: green) and a GFP-negative dendrite (a presumptive pre-existing GC: blue). Arrows point to the postsynaptic density. Note that the synapse onto the GFP-positive profile has a complex morphology and shows clear perforation. Scale bar = 50 nm. Click here for file
