# Supplementary figures and images for: Dynamic development of the first synapse impinging on adult-born neurons in the olfactory bulb circuit
Source: Neural Syst Circuits. 2011 Feb 1;1:6. doi: 10.1186/2042-1001-1-6 (PMC3278389; doi:10.1186/2042-1001-1-6)

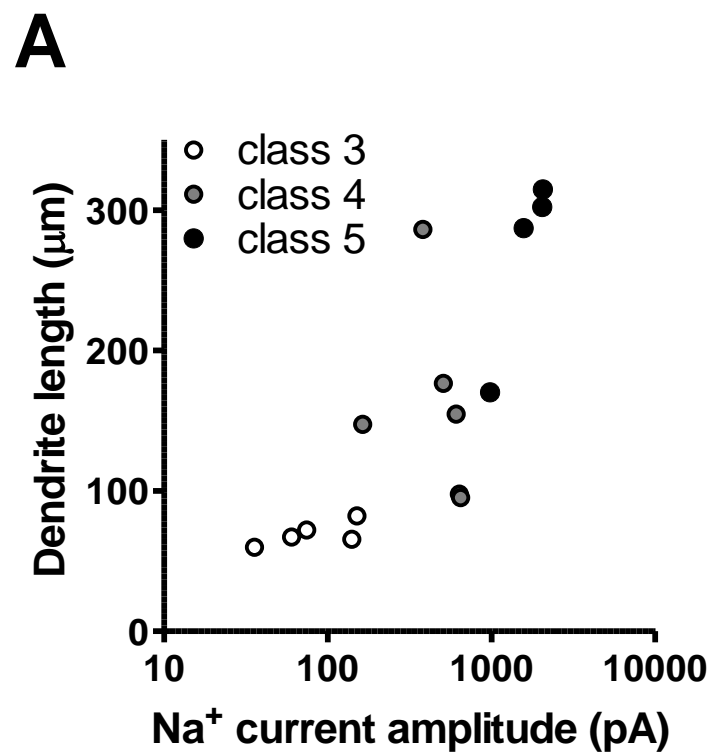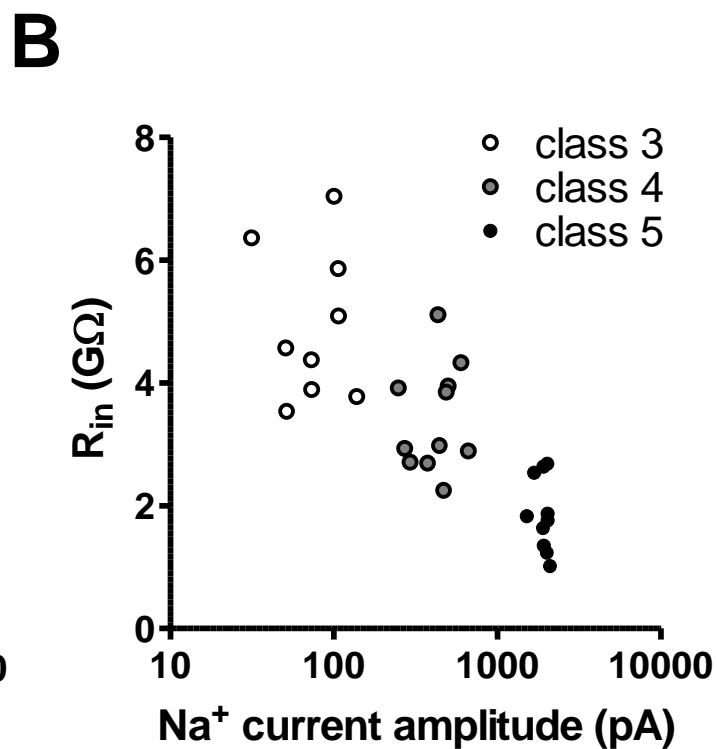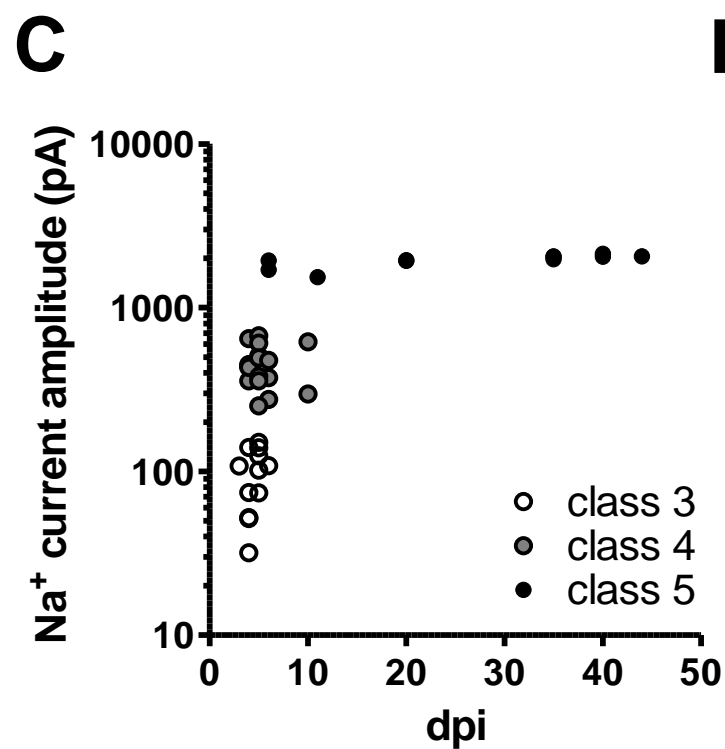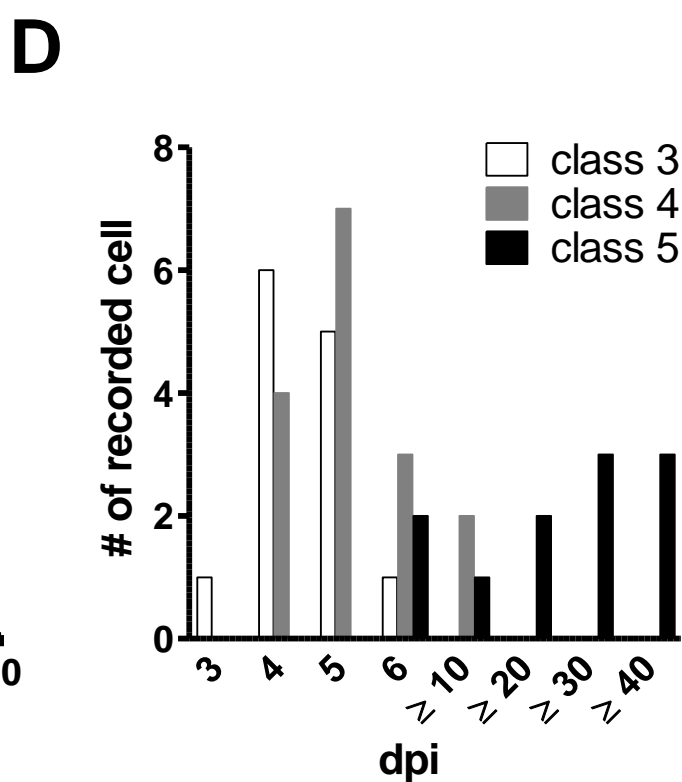

Supplemental Figure 1

Supplement: Additional file 1 — Supplementary Figure 1: Classification of developing adult-generated granule cells (GCs). (A) Relationship between length of apical dendrite and maximal Na+ current evoked by a depolarizing step pulse. Adult-born GCs were classified according to Na+ current amplitude, with white, gray and black circles representing classes 3, 4 and 5, respectively. Apical dendrites elongated during maturation. (B) Inverse correlation between the input membrane resistance (Rin) and maximum Na+ current amplitude evoked by a depolarizing step pulse. (C) Plots of maximum Na+ current amplitude evoked by a depolarizing voltage step versus days post-injection (dpi) of virus. (D) Records from various newborn GCs after viral injection. Note the absence of clear boundaries among classes. [file 2042-1001-1-6-S1.PDF]

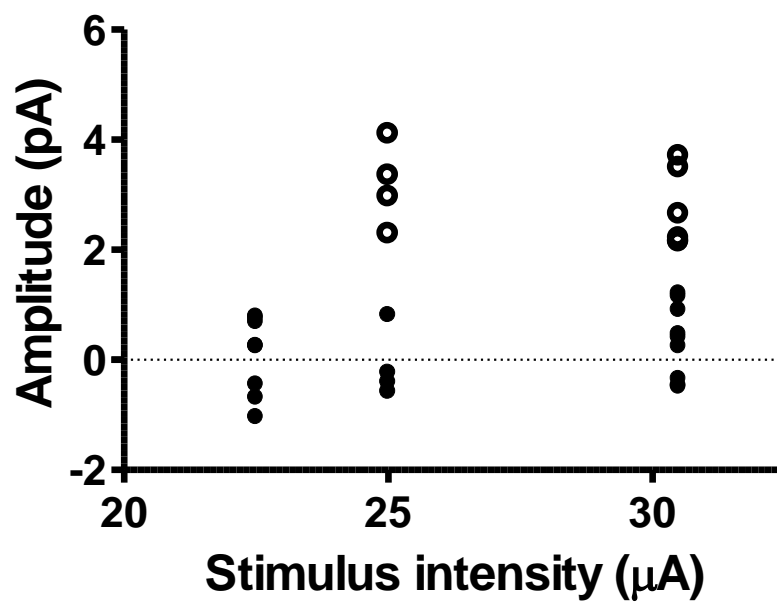

Supplemental Figure 2

Supplement: Additional file 2 — Supplementary Figure 2: Proximal synaptic responses evoked by minimal stimulation. Gradually increasing stimulus intensity abruptly evoked events in an all-or-none manner. Open and closed circles indicate response success and failure, respectively. Unitary response amplitude was confirmed to be constant by a small increase in the stimulus intensity. [file 2042-1001-1-6-S2.PDF]

**A**

class 3

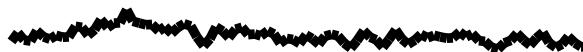

class 4

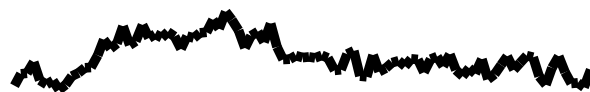

class 5

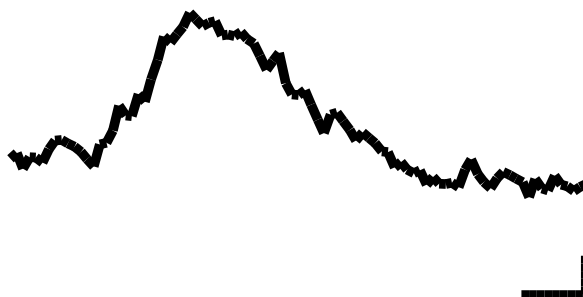**B**

AMPA

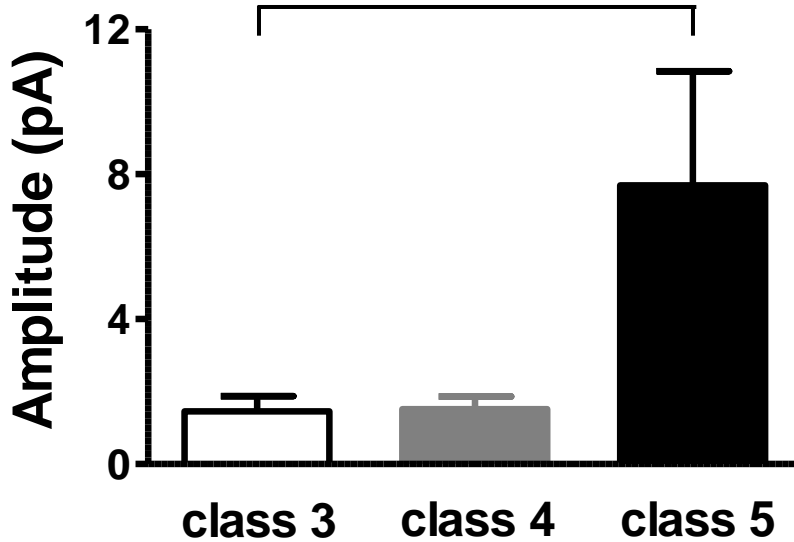

Supplement: Additional file 3 — Supplementary Figure 3: Developmental change of 3-hydroxy-5-methyl-4-isoxazolepropionic acid receptor (AMPAR)-mediated outward currents at depolarized membrane potentials. (A) Typical traces. After AMPAR-mediated and N-methyl D-aspartate receptor (NMDAR)-mediated currents were recorded at the holding potential of +40 mV, 2,3-dioxo-6-nitro-1,2,3,4-tetrahydrobenzo[f]quinoxaline-7-sulfonamide (NBQX) was applied to obtain NMDAR-mediated excitatory postsynaptic currents (EPSCs). Traces were derived from the subtraction of NMDAR-mediated EPSCs from AMPAR-mediated and NMDAR-mediated currents. Scale bar = 1 ms and 1 pA. (B) The amplitude of AMPAR-mediated EPSCs was much higher in class 5 than in class 3 cells (class 3: 1.43 ± 0.43; class 4: 1.52 ± 0.34; class 5: 7.69 ± 3.15) (class 3 versus class 5, *P < 0.05) (class 3: six slices, six mice; class 4: eight slices, eight mice; class 5: four slices, four mice). [file 2042-1001-1-6-S3.PDF]

**A**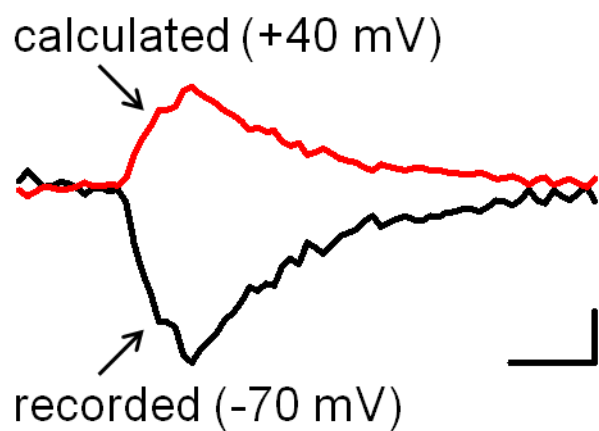**B**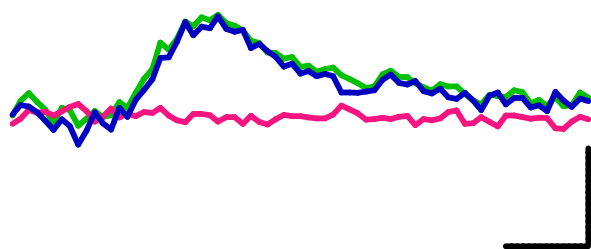**C**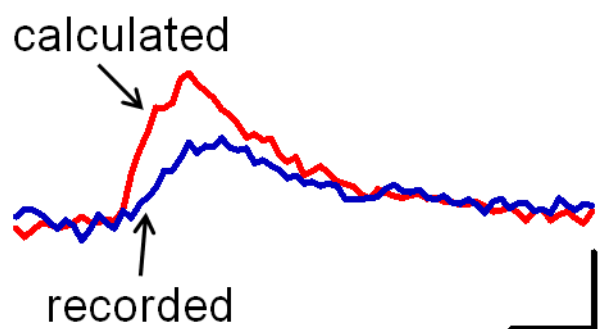**D**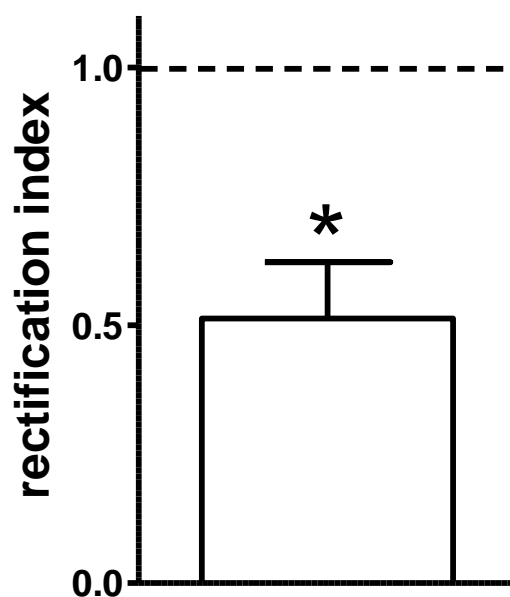

Supplemental Figure 4

Supplement: Additional file 4 — Supplementary Figure 4: Ca2+-permeable AMPARs in class 3 GCs. (A) (Black) AMPAR-mediated EPSCs at the holding potential of -70 mV (red). This trace shows predicted data, obtained by inversion of the trace shown in black and multiplication by four-sevenths. (B) (Green) AMPAR-mediated and NMDAR-mediated EPSCs at the holding potential of +40 mV. (Pink) NMDAR-mediated EPSCs at the same holding potential. This current was recorded in the presence of NBQX. (Blue) AMPAR-mediated current at the holding potential of +40 mV. This experimental trace was generated by subtracting the NMDAR-mediated component from both receptor-mediated currents (Green minus pink). (C) Comparison between the predicted and experimental traces. (A-C) Scale bar = 1 ms and 2 pA. (D) The maximum amplitude of the trace obtained from dividing experimental values by predicted values. If AMPAR-mediated EPSCs in class 3 GCs are mediated by Ca2+-impermeable AMPARs, this ratio would be equal to 1 (dashed line), given the linear current-voltage relationship of Ca2+-impermeable AMPAR. The experimental values obtained were significantly lower than the predicted values (class 3: six slices; *P < 0.05). [file 2042-1001-1-6-S4.PDF]

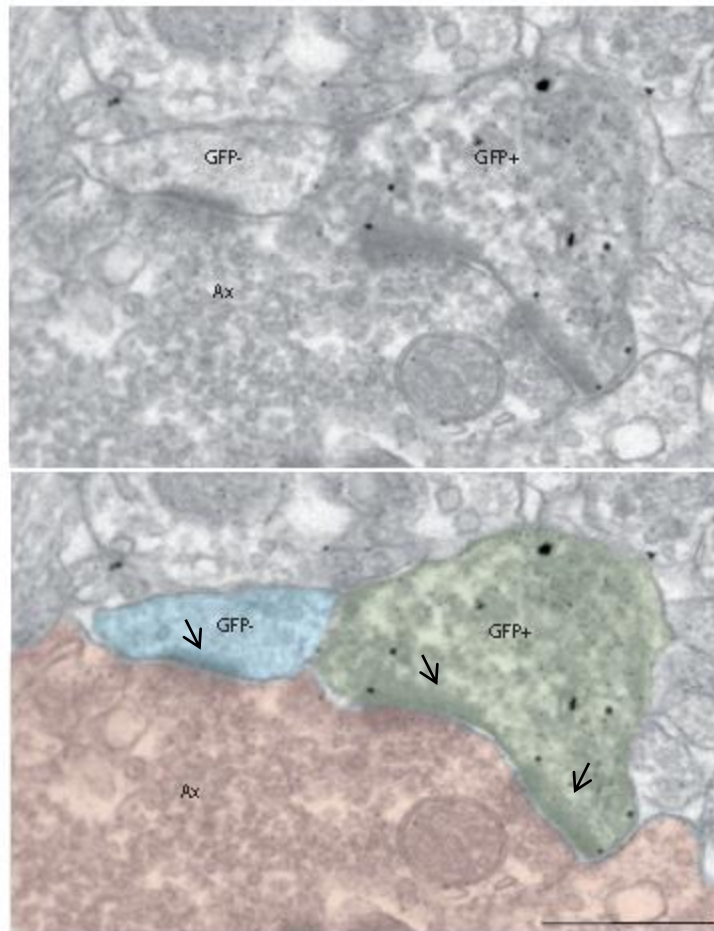

Supplemental Figure 5

Supplement: Additional file 5 — Supplementary Figure 5: Axon terminals make contacts with both adult-born and pre-existing GCs. A large axon terminal (Ax: red) makes two synapses with a GFP-positive dendrite (GC at 7 days post-injection: green) and a GFP-negative dendrite (a presumptive pre-existing GC: blue). Arrows point to the postsynaptic density. Note that the synapse onto the GFP-positive profile has a complex morphology and shows clear perforation. Scale bar = 50 nm. [file 2042-1001-1-6-S5.PDF]
